# Ballistic Performance and Energy Dissipation Mechanisms of Epoxy Composites Reinforced with Raffia Fabric Under 9 mm Impact

**DOI:** 10.3390/polym18080903

**Published:** 2026-04-08

**Authors:** Douglas Santos Silva, Raí Felipe Pereira Junio, Elias Matias Bentes, Thomaz Jacintho Lopes, Belayne Zanini Marchi, Sergio Neves Monteiro

**Affiliations:** Department of Materials Science, Military Institute of Engineering—IME, Praça General Tibúrcio, 80, Praia Vermelha, Urca, Rio de Janeiro CEP 22290-270, RJ, Brazil; raivsjfelipe@ime.eb.br (R.F.P.J.); elias.bentes@ime.eb.br (E.M.B.); thomazjlopes@gmail.com (T.J.L.); belayne@ime.eb.br (B.Z.M.); sergio.neves@ime.eb.br (S.N.M.)

**Keywords:** ballistic performance, epoxy composites, raffia fiber, energy absorption, natural fiber reinforcement

## Abstract

This study investigates the ballistic performance and energy dissipation mechanisms of epoxy composites reinforced with raffia fabric at fiber volume fractions of 10%, 20%, and 30% under 9 mm full metal jacket projectile impact. Ballistics tests were conducted to determine impact and residual velocities, absorbed energy, absorption efficiency, equivalent ballistic limit, and momentum reduction. All tests were performed at similar impact velocities (≈433 m/s), corresponding to an incident energy of approximately 750 J. The results revealed a clear inverse relationship between raffia content and energy absorption capability. The ER10 composite exhibited the highest performance, with an absorbed energy of 176.7 ± 9.7 J, absorption efficiency of 23.5 ± 0.9%, and momentum reduction of 0.1253 ± 0.0053. Increasing the fiber fraction to 20% (ER20) and 30% (ER30) led to progressive reductions in absorbed energy to 119.7 ± 2.7 J and 77.7 ± 9.0 J, with efficiencies of 15.95 ± 0.26% and 10.30 ± 1.12%, respectively. The residual velocity increased from 379.3 ± 2.5 m/s (ER10) to 397.0 ± 2.1 m/s (ER20) and 411.1 ± 1.6 m/s (ER30). One-way ANOVA detected statistically significant differences in absorbed energy and absorption efficiency among the different fiber volume fractions (*p* < 0.001). The results demonstrate a trade-off between stiffness and toughness and indicate that raffia-reinforced composites can play complementary roles in sustainable multilayered armor systems.

## 1. Introduction

The growing demand for lightweight, efficient, and cost-effective ballistic protection systems has intensified research into polymer matrix composites capable of dissipating high levels of impact energy. Conventional armor solutions commonly rely on high-performance synthetic reinforcements, such as aramid fibers, ultra-high-molecular-weight polyethylene (UHMWPE), and glass fibers, combined with polymeric matrices to achieve high strength-to-weight ratios and reliable penetration resistance. Despite their well-established effectiveness, these materials are often associated with high production costs and relatively high energy demand during manufacturing, whereas natural lignocellulosic fibers generally present lower environmental impact and reduced processing energy requirements [1,2]. Consequently, increasing attention has been directed toward alternative reinforcement strategies that combine adequate ballistic performance with improved sustainability and resource efficiency [3,4,5,6,7].

In this context, natural lignocellulosic fibers (NLFs) have emerged as promising candidates for energy-dissipating components in protective structures. Their low density, renewability, reduced abrasiveness, and favorable specific mechanical properties have enabled their application as intermediate or backing layers in multilayered armor systems (MAS). Several studies have demonstrated that NLF-reinforced polymer composites, such as those incorporating jute, sisal, hemp, curaua, and carnauba fibers, can exhibit competitive ballistic responses when properly designed, particularly in terms of energy absorption efficiency and weight reduction [8]. These characteristics make natural fiber composites especially attractive for hybrid armor concepts, in which different layers perform complementary mechanical functions.

Among the various natural lignocellulosic fibers (NLFs) investigated to date, raffia fiber (*Raphia vinifera*), widely available in the Amazon region, remains comparatively underexplored for ballistic applications. Previous studies have reported its favorable thermochemical stability, low density, and suitable structural characteristics for use as reinforcement in polymer composites [9]. More recent investigations have demonstrated that raffia fabric–reinforced epoxy composites can provide meaningful ballistic resistance under impact, both as standalone targets and as intermediate layers in multilayered armor systems [10,11,12].

In particular, previous works by the present research group investigated the ballistic behavior of ER10, ER20, and ER30 laminates under handgun-level impact conditions and demonstrated the feasibility of these materials for energy-dissipating applications. However, those studies were primarily limited to global ballistic indicators—such as absorbed energy and residual velocity—and qualitative descriptions of damage mechanisms, without exploring the dynamic aspects of the impact event or providing a statistically validated comparison between different fiber volume fractions under strictly controlled conditions.

It is important to note that previous studies by the authors also involved high-energy ballistic conditions using 7.62 mm ammunition and multilayered armor systems (MAS), focusing on penetration resistance and structural integrity. In contrast, the present study investigates the ballistic response under lower-energy impact conditions using 9 mm ammunition, enabling a detailed analysis of energy absorption mechanisms, dynamic response, and statistical behavior of the composite system.

In contrast, the present study introduces a quantitatively driven analytical framework for evaluating the ballistic response of raffia-reinforced composites. Specifically, this work incorporates time-resolved projectile velocity measurements obtained via Doppler radar, enabling a more detailed interpretation of the impact process. In addition, the ballistic performance is systematically decomposed into energy absorption efficiency and projectile momentum reduction, allowing a deeper understanding of the mechanisms governing energy dissipation. Furthermore, a rigorous statistical approach (one-way ANOVA followed by Tukey’s post hoc test) is employed to assess the significance of the influence of fiber volume fraction on ballistic performance metrics under equivalent impact conditions.

Therefore, the present work advances beyond previous studies by providing a statistically validated and energy-based interpretation of the ballistic behavior of raffia fabric–reinforced epoxy composites, offering new insights into the role of fiber volume fraction in governing energy dissipation mechanisms in polymer matrix composites.

In particular, the quantitative influence of raffia fabric volume fraction on specific energy-based ballistic metrics has not yet been systematically analyzed. Existing studies often emphasize absolute absorbed energy values, without fully addressing efficiency-related parameters such as the fraction of incident energy dissipated, momentum reduction, or the statistical robustness of the observed trends. Moreover, comparisons are frequently performed under different impact severities, projectile velocities, or target configurations, making it difficult to establish clear design guidelines regarding the optimal fiber content for energy dissipation versus structural integrity. As a result, it remains unclear how the raffia fabric volume fraction quantitatively influences specific ballistic performance parameters. In particular, it remains uncertain whether increasing the raffia fiber volume fraction necessarily enhances ballistic performance, or whether excessive reinforcement may compromise the energy dissipation capability, potentially due to microstructural limitations such as incomplete matrix impregnation and reduced fiber–matrix interaction, which may affect the efficiency of stress transfer and energy dissipation mechanisms during impact [1].

Despite the growing body of literature on natural lignocellulosic fiber (NLF)-reinforced composites for ballistic applications, most studies have focused on well-established fibers such as jute, sisal, hemp, and curaua, with comparatively limited attention given to raffia fibers. In addition, existing investigations often emphasize absolute absorbed energy or penetration resistance, with less focus on normalized energy-based metrics and their statistical validation under controlled impact conditions.

In this context, the present study advances the current state of the art by providing a systematic and statistically supported evaluation of the ballistic performance of raffia fabric–reinforced epoxy composites. Unlike previous works, this study integrates multiple energy-based and dynamic parameters, including absorption efficiency, momentum reduction, and velocity–time response, all obtained under equivalent impact conditions. This approach enables a more comprehensive understanding of the energy dissipation mechanisms governing ballistic performance.

Furthermore, the results reveal a non-intuitive trend in which increasing the fiber volume fraction does not improve, but rather reduces the energy absorption capability of the composite under the processing conditions adopted. This finding provides important design-oriented insight that contrasts with the commonly assumed positive correlation between reinforcement content and mechanical performance in fiber-reinforced composites. Therefore, this work contributes not only to the characterization of raffia-based composites but also to the broader understanding of the role of fiber content in the ballistic response of sustainable composite materials.

Therefore, this study aims to systematically evaluate the ballistic performance of epoxy composites reinforced with raffia fabric at fiber volume fractions of 10%, 20%, and 30% under 9 mm full metal jacket projectile impact. Ballistics tests were conducted at comparable impact velocities (≈433 m/s), corresponding to an incident energy of approximately 750 J, allowing a consistent comparison among the different composite configurations. The impact and residual velocities were measured using Doppler radar, enabling the determination of absorbed energy, energy absorption efficiency, equivalent ballistic limit velocity, and momentum reduction. To ensure the reliability and significance of the observed trends, the experimental results were supported by a robust statistical analysis based on one-way ANOVA and Tukey’s post hoc test.

By combining energy-based, kinematic, and statistical analyses under identical impact conditions, this work provides design-oriented evidence that higher natural fiber contents do not necessarily translate into improved ballistic efficiency. The results establish a clear hierarchy of performance among the investigated fiber volume fractions and offer practical guidelines for the rational use of raffia-reinforced epoxy composites in sustainable ballistic protection systems, particularly in functionally graded or multilayered armor architectures.

## 2. Materials and Methods

### 2.1. Materials

An epoxy resin based on diglycidyl ether of bisphenol A (DGEBA) was used as the polymer matrix. According to the manufacturer’s technical datasheet, the resin presents an epoxy equivalent weight (EEW) of approximately 185–192 g·eq^−1^, which is typical for DGEBA-based systems and governs the stoichiometric ratio with the curing agent as well as the resulting crosslink density of the cured network. The resin was cured using a commercial amine-based hardener following the proportion recommended by the manufacturer. The resin was combined with a triethylenetetramine (TETA) hardener at a stoichiometric ratio of 100:13 by weight, as recommended by the supplier. According to previous studies, the cured DGEBA system exhibits a nominal density of 1.11 g/cm^3^ [13]. Both components were supplied by Dow Chemical (São Paulo, Brazil) and distributed locally by Resin Epoxy Ltd. (Rio de Janeiro, Brazil).

**Figure 1:**
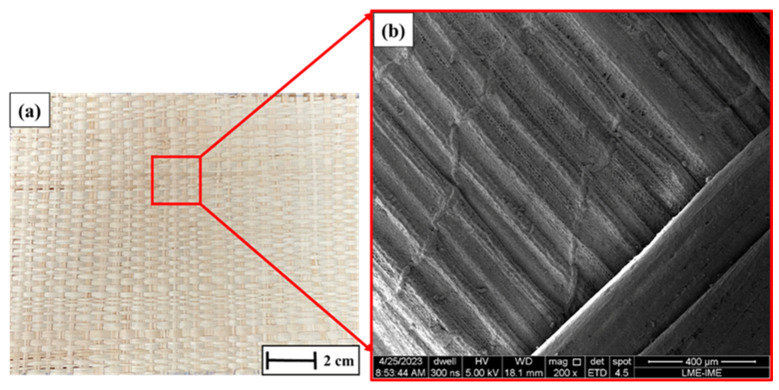
Morphological characterization of the raffia fabric (*Raphia vinifera*): (**a**) macroscopic view of the plain-woven textile architecture (scale bar: 2 cm); (**b**) SEM micrograph showing the longitudinal fiber morphology and cellular structure (200× magnification, scale bar: 400 µm).

The raffia fibers used as reinforcement were obtained from the *Raphia vinifera* species and supplied in the form of plain-woven fabrics sourced from the local market in Belém, Brazil. The raffia reinforcement used in this study consists of a bidirectional woven fabric, in which the fibers are oriented along two orthogonal directions (0°/90°). This woven architecture provides in-plane reinforcement and contributes to load distribution during impact. All fabric layers were stacked maintaining the same orientation throughout the laminate, without intentional rotation between successive plies. This configuration ensures a consistent reinforcement architecture and allows a direct evaluation of the effect of fiber volume fraction on the ballistic performance. The intrinsic density of raffia fibers is reported in the literature to be approximately 0.95 g·cm^−3^, which is consistent with values typically observed for natural lignocellulosic fibers [9]. In the present study, this value was adopted as the fiber density for the estimation of the composite volume fractions. It should be noted that this parameter corresponds to the intrinsic density of the fibers rather than the density of the woven fabric, which depends on parameters such as fabric architecture, areal density, and thickness. The raffia reinforcement used in this study consists of a woven fabric, whose architecture plays an important role in the mechanical and ballistic behavior of the composite. The fabric is characterized by its weave pattern, yarn distribution, and areal density, which influence fiber packing, load transfer, and energy dissipation mechanisms. Although natural variability is inherent to lignocellulosic materials, the raffia fabric employed can be described as a bidirectional woven structure, with interlaced yarns providing in-plane reinforcement. The areal weight and yarn density contribute to the overall fiber volume fraction and affect the impregnation quality and interfacial bonding with the epoxy matrix. These parameters are particularly relevant in ballistic applications, as they influence deformation mechanisms such as yarn mobility, frictional sliding, and delamination resistance within the composite structure. Figure 1 presents the morphology of the raffia fabric used in this study.

### 2.2. Methods

#### 2.2.1. Composite Manufacturing

Prior to lamination, the as-received raffia fabrics were cut into rectangular sheets measuring 120 mm × 150 mm and subsequently oven-dried at 60 °C for 24 h to eliminate residual moisture. The dried fabrics were then placed inside a metallic mold designed to produce plates with final dimensions of 150 mm × 120 mm × 12 mm. To facilitate demolding, the inner mold surfaces were coated with a thin layer of silicone-based release agent.

**Figure 2:**
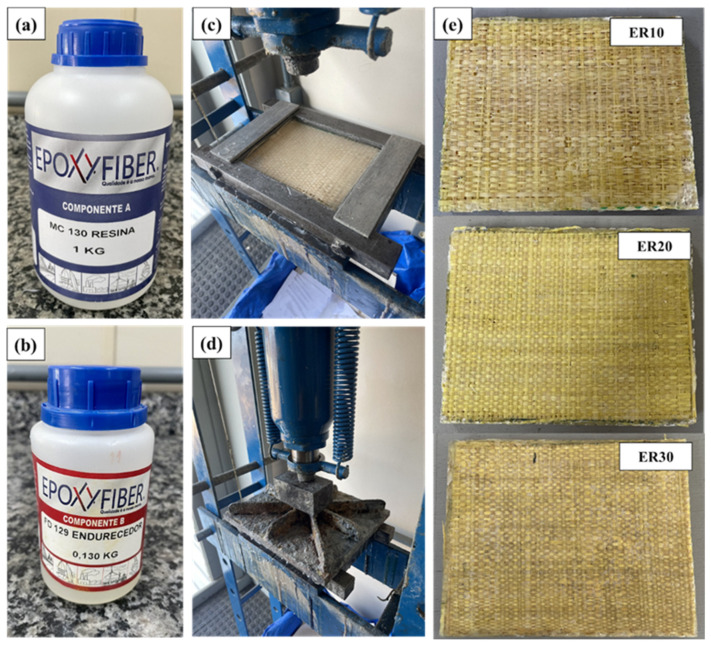
Composite manufacturing stages and final laminates: (**a**) epoxy resin; (**b**) hardener; (**c**) mold with raffia fabric; (**d**) compression molding; (**e**) consolidated laminates ER10 (10 vol.%), ER20 (20 vol.%), and ER30 (30 vol.%).

The raffia fabrics were carefully arranged within the mold cavity and thoroughly impregnated with the epoxy–hardener mixture. After mold closure, a compressive load of approximately 5 tons was applied and maintained for 24 h to ensure adequate consolidation and fiber wet-out. The curing process was carried out at ambient temperature (~25 °C). Composite plates were produced with three nominal fiber volume fractions of 10, 20, and 30 vol%, corresponding to laminates reinforced with 6, 12, and 18 layers of raffia fabric, respectively. Each raffia fabric layer presented an average mass of approximately 3.3 g for the plate dimensions adopted in this study. Based on the measured mass of each raffia fabric layer (approximately 3.3 g) and the nominal layer dimensions (120 mm × 150 mm), the areal density of the raffia fabric was estimated as approximately 183 g/m^2^. This value remained constant for all laminate configurations, as it is an intrinsic property of the fabric. The different fiber volume fractions (ER10, ER20, and ER30) were therefore achieved by varying the number of fabric layers. The fiber volume fraction (V_f_) was estimated based on the ratio between the total fiber volume and the composite volume. The total fiber volume was obtained from the measured mass of raffia reinforcement and the intrinsic fiber density (ρ_f_ ≈ 0.95 g·cm^−3^), while the composite volume was determined from the mold dimensions and the final laminate thickness measured after curing using a digital caliper. Accordingly, the fiber volume fraction was calculated as: V_f_ = mf/(ρ_f_ · V_c_), where mf is the total fiber mass, ρ_f_ is the fiber density, and V_c_ is the composite volume. Additionally, the apparent density of the cured laminates was estimated from the measured mass and geometric volume (ρ_c_ = m_c_/V_c_), providing an indirect validation of the calculated fiber volume fractions. The obtained density values were found to be consistent with typical ranges reported for natural fiber–reinforced epoxy composites, supporting the reliability of the nominal V_f_ values adopted in this study. Although local variations associated with fabric architecture (e.g., crimp and porosity) may affect the exact fiber distribution, this approach provides a consistent and reproducible estimation of nominal fiber volume fraction for comparative purposes. The thickness of the specimens was measured at multiple points, resulting in an average value of approximately 10 mm with a variation within ±0.2 mm. This low variability indicates a high level of thickness uniformity, ensured by the rigid mold constraints and controlled curing conditions. As a result, the influence of thickness variation on the ballistic performance can be considered negligible. A neat epoxy plate (0 vol% fiber) was not included as a ballistic target in this study because unreinforced epoxy exhibits brittle fracture and catastrophic fragmentation under high-velocity impact. This behavior prevents the establishment of stable perforation conditions and does not allow reliable measurement of key ballistic parameters such as residual velocity and absorbed energy. In contrast, fiber-reinforced laminates enable energy dissipation through mechanisms such as fiber bridging, pull-out, delamination, and progressive damage, which are essential for meaningful ballistic performance evaluation. Therefore, the present study focuses on reinforced configurations that are representative of structurally viable materials for ballistic protection applications. Figure 2 illustrates the appearance of the fabricated laminates.

#### 2.2.2. Stand-Alone Ballistics Tests

**Figure 3:**
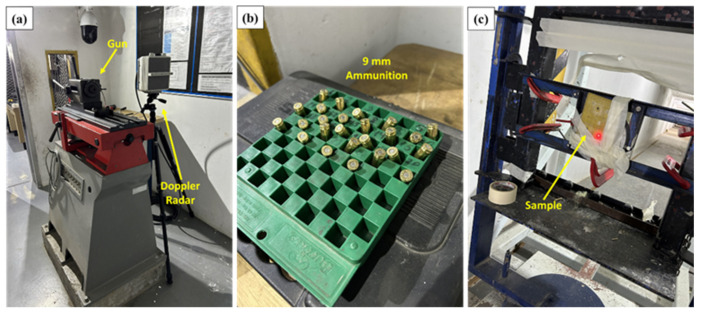
Experimental setup for the ballistics tests: (**a**) firing system with 9 mm gun and Doppler radar for velocity measurement; (**b**) 9 mm full metal jacket ammunition used in the experiments; (**c**) target holder with composite specimen positioned for impact testing.

Ballistic experiments were carried out at the Brazilian Army Assessment Center (CAEx), Rio de Janeiro (see Figure 3). For each material condition, five shots were performed. The firing system comprised the High-Pressure Instrumentation (HPI) barrel mounted so that the target was positioned 5 m downrange. A B290 rig equipped with a laser aiming device was used to ensure repeatable alignment with the specimen. The projectile mass used for the energy calculations was 8.0 g (0.008 kg), corresponding to the nominal mass specified by the manufacturer for the 9 mm full metal jacket ammunition employed in the ballistics tests. This value was adopted as the representative projectile mass in the kinetic energy calculations, since the mass variation among cartridges of the same lot is typically minimal compared to the variability observed in projectile velocity measurements. The projectile trajectory was aligned normal to the plate surface (nominal 0° incidence). The target holder was positioned coaxially with the firearm barrel to ensure perpendicular impact. The angular deviation between the projectile trajectory and the target surface was maintained within an estimated tolerance of approximately ±1°, verified through geometric alignment of the ballistic setup and careful positioning of the target prior to each shot. Projectile velocities before and after perforation were recorded using a Weibel SL-520P Doppler radar (Weibel Scientific A/S, Allerød, Denmark). The velocity data were processed using WinDopp software (Weibel Scientific A/S, Allerød, Denmark). 

The projectile velocities (impact and residual) were measured using a Doppler radar system, which provides continuous velocity tracking during the ballistic event. The accuracy of Doppler radar measurements typically lies within a small percentage range of the measured value, depending on calibration conditions and signal quality. In the present study, the variability associated with velocity measurements is reflected in the standard deviations reported for impact and residual velocities, as well as in the derived ballistic parameters such as absorbed energy and equivalent ballistic limit velocity. These statistical variations inherently account for both experimental scatter and measurement uncertainties. Therefore, the reported results incorporate the combined effects of material response variability and instrumental uncertainty, ensuring a reliable representation of the ballistic performance of the tested composites.

It is important to note that all ballistics tests in this study were conducted at impact velocities above the ballistic limit, resulting in complete perforation of the targets. This approach was intentionally adopted to evaluate the energy absorption capacity and damage mechanisms of the composites under high-severity impact conditions, which are representative of real ballistic threats. Although tests performed at velocities close to the ballistic limit can provide more detailed insight into the transition between partial and complete penetration, the focus of the present work is on comparative performance under full perforation conditions, where differences in energy dissipation and structural integrity become more evident. Future studies may explore impact conditions near the ballistic limit to further investigate the penetration resistance and threshold behavior of raffia-reinforced composites.

In the present study, the ballistic performance was evaluated using 9 mm full metal jacket (FMJ) ammunition, which provides a standardized and widely adopted impact condition for comparative analysis. This type of projectile was selected due to its well-defined geometry, mass, and impact behavior, allowing for consistent assessment of energy absorption and perforation response. It is acknowledged that the ballistic response of composite materials can vary significantly with projectile characteristics, including shape, mass, hardness, and nose geometry (e.g., pointed, armor-piercing, or fragment-simulating projectiles). These variations influence penetration mechanisms, stress distribution, and damage evolution within the target. Therefore, the results presented in this study should be interpreted within the context of 9 mm FMJ impact conditions. Future investigations may consider different projectile types and threat levels to provide a more comprehensive assessment of the ballistic performance of raffia-reinforced composites.

#### 2.2.3. Ballistic Data Analysis

The ballistic performance of the raffia/epoxy composite plates was assessed through the analysis of their kinetic response under 9 mm projectile impact. The incident (V_i_) and residual (V_r_) velocities of the projectiles were recorded using a Weibel SL-520P Doppler radar, allowing the determination of the energy transfer, momentum variation, and the operational ballistic limit for each fiber volume fraction (10, 20, and 30 vol%).

All calculations were performed assuming a projectile mass of 8 g (0.008 kg). The incident (E_in_) and residual (E_res_) kinetic energies were first determined according to Equations (1) and (2):

(1) $$E_{in}=\frac{1}{2}mV_{i^{2}}$$

(2) $$E_{res}=\frac{1}{2}mV_{r^{2}}$$

The difference between these two quantities corresponds to the absorbed kinetic energy (E_abs_), representing the energy dissipated through mechanisms such as matrix cracking, fiber rupture, delamination, and frictional losses during impact, as Equation (3):

(3) $$E_{abs}=E_{in}-E_{res}=\frac{1}{2}m\left(V_{i^{2}-}V_{r^{2}}\right)$$

To compare the ability of each composite to attenuate the projectile’s kinetic energy, the energy absorption efficiency (ηE) was calculated as the ratio between the absorbed and the incident energies, as given by Equation (4):

(4) $$\eta E=\frac{E_{abs}}{E_{in}}\times 100=\left(1-\frac{V_{r^{2}}}{V_{i^{2}}}\right)\times 100$$

In addition to energy parameters, the momentum reduction (Δp) was determined to quantify the impulse transmitted through the composite plate, following Equation (5):

(5) $$∆p=m\left(V_{i}-V_{r}\right)\;\mathrm{and}\;\%∆p=\frac{V_{i}-V_{r}}{V_{i}}\times 100$$

Since all tests were conducted at velocities well above the experimental ballistic limit, resulting in complete perforation of the targets, the ballistic limit velocity was not determined experimentally through a series of partial and complete penetration tests. Instead, an equivalent ballistic limit velocity (V_L_) was estimated from the measured velocities assuming a simplified energy-based penetration model, in which the residual velocity is related to the impact velocity by Equation (6):

(6) $${V_{r}}^{2}={V_{i}}^{2}-{V_{L}}^{2}$$

The ballistic response of the targets was evaluated using the relationship between impact velocity (V_i_) and residual velocity (V_r_). The parameter commonly referred to as the limit velocity (V_L_) was estimated according to Equation (7):

(7) $$V_{L}=\sqrt{{V_{i}}^{2}-{V_{r}}^{2}}$$

This approach, commonly adopted in ballistic studies when full perforation occurs, provides a comparative metric to evaluate the relative resistance of materials under identical impact conditions [2]. The formulation is derived from the residual velocity model originally proposed by Recht and Ipson, which relates impact and residual velocities through energy balance during projectile perforation. It is important to emphasize that V_L_ represents a characteristic velocity parameter associated with energy dissipation during perforation rather than a true ballistic limit, since a strict ballistic limit requires the condition V_r_ = 0, typically determined through velocity variation tests.

The use of this parameter allows a consistent comparison between the ER10, ER20, and ER30 composites, highlighting the influence of raffia fabric volume fraction on the energy dissipation capability and penetration resistance of the laminates.

It is important to note that the equivalent ballistic limit velocity (V_L_) obtained in this study is not directly measured, but rather estimated from an energy-based analytical model using the measured impact (V_i_) and residual (V_r_) velocities. As a result, the calculated V_L_ inherently carries uncertainty associated with the experimental variability of velocity measurements and the assumptions of the adopted model.

In particular, uncertainties in Vi and V_r_ propagate through the calculation of V_L_, and the simplified energy balance formulation does not explicitly account for complex phenomena such as projectile deformation, frictional losses, or non-uniform damage evolution within the laminate. Therefore, the reported V_L_ values should be interpreted as comparative indicators of ballistic performance under consistent test conditions, rather than absolute material properties.

Despite these limitations, this approach is widely adopted in ballistic studies involving complete perforation, as it enables a consistent and physically meaningful comparison between different material configurations tested under similar conditions.

#### 2.2.4. Projectile Velocity–Time History

The velocity–time history of the projectile during impact and perforation was obtained using a Doppler radar system. This technique enables continuous measurement of the projectile velocity along its trajectory, capturing the deceleration process as the projectile interacts with the target and subsequently propagates through free flight after perforation.

The recorded curves exhibit three distinct stages: (i) a pre-impact region characterized by an approximately constant velocity corresponding to the impact velocity (V_i_); (ii) a rapid deceleration stage associated with the projectile–target interaction and energy dissipation mechanisms within the laminate; and (iii) a post-perforation region, in which the projectile exits the target with a residual velocity (V_r_) and undergoes gradual deceleration due to aerodynamic drag.

Although the Doppler radar measures the projectile velocity along its flight path, the sharp velocity drop observed during the interaction stage provides qualitative insight into the energy dissipation mechanisms occurring within the composite. Differences in the slope and magnitude of this deceleration among the ER10, ER20, and ER30 laminates reflect variations in their resistance to penetration and capacity to absorb kinetic energy.

It should be emphasized that the velocity histories reported in this study correspond to the longitudinal velocity of the projectile, not to the radial propagation velocity of damage within the composite laminate. Therefore, the present analysis focuses on the projectile response during impact rather than on in-plane damage propagation phenomena.

#### 2.2.5. Statistical Analysis

The statistical analysis was performed to evaluate the influence of raffia fabric volume fraction on the ballistic performance of the composites. The datasets obtained for absorbed energy (E_abs_) and energy absorption efficiency were analyzed using one-way analysis of variance (ANOVA) with a significance level of α = 0.05.

Prior to performing ANOVA, the assumptions required for parametric statistical analysis were verified. The Shapiro–Wilk test was applied to assess the normality of the datasets, while Levene’s test was used to evaluate the homogeneity of variances among the experimental groups (ER10, ER20, and ER30). The corresponding test statistics and *p*-values are presented in Table 1.

The Shapiro–Wilk test indicated that both variables followed normal distributions. Specifically, the absorbed energy dataset yielded W = 0.965 (*p* = 0.78), while the absorption efficiency dataset yielded W = 0.961 (*p* = 0.73). Likewise, Levene’s test confirmed the homogeneity of variances among the groups, with *p* = 0.36 for absorbed energy and *p* = 0.41 for absorption efficiency. Since all *p*-values were greater than 0.05, the assumptions of normality and homoscedasticity required for ANOVA were satisfied.

After confirming these assumptions, one-way ANOVA was applied to determine whether statistically significant differences existed among the composite configurations. When significant differences were detected, Tukey’s honestly significant difference (HSD) post hoc test was performed to identify pairwise differences between the group means.

All statistical results are reported as mean values accompanied by their respective standard deviations, calculated from five independent ballistics tests for each composite configuration.

#### 2.2.6. Post-Impact Damage Documentation

After ballistic testing, all specimens were visually inspected and photographed to document the macroscopic damage patterns on both front and rear faces. Images were acquired using a high-resolution digital camera under controlled lighting conditions. The analysis focused on qualitative assessment of perforation morphology, delamination extension, fiber rupture, and matrix cracking patterns. No microscopic (SEM) analysis was conducted in the present study.

## 3. Results and Discussion

All ballistic results presented in this section are based on five independent shots per configuration, as described in Section 2.2.2, ensuring statistical reliability and reproducibility of the measured parameters. All results are reported as mean values accompanied by standard deviation (mean ± SD), and were statistically validated through normality (Shapiro–Wilk), homogeneity of variance (Levene’s test), one-way analysis of variance (ANOVA), and Tukey’s post hoc test, as described in Section 2.2.5.

### 3.1. Ballistic Response: Impact and Residual Velocities

The average impact and residual velocities obtained for the ER10, ER20, and ER30 laminates are summarized in Table 2.

It should be noted that the small differences observed in the standard deviations of the impact velocities among the experimental groups are not related to the composite configuration. The impact velocity is determined by the ballistic characteristics of the ammunition and firearm rather than by the target material. Therefore, the variations observed in Table 2 primarily reflect the natural dispersion associated with commercially manufactured ammunition and the limited number of shots performed in each group (n = 5). The slightly lower standard deviation observed for the ER30 condition is thus attributed to statistical variability of the projectile velocities rather than to any systematic effect related to fiber content or target response.

The results obtained in the ballistic test with 9 mm projectiles in epoxy composites reinforced with raffia fabric show significant differences in the behavior of the materials according to the volumetric fraction of incorporated fiber. The average impact velocities, close to 433 m/s in all samples, with standard deviations of less than 1%, indicate high reproducibility and experimental control, which allows attributing the variations observed in residual velocities exclusively to the ballistic performance of the composites and not to fluctuations in the firing system.

Analysis of residual velocities reveals a clear increasing trend as fiber content increases: the average value went from 379.3 m/s for the ER10 composite (10% raffia) to 397.0 m/s in ER20 (20%) and reached 411.1 m/s in ER30 (30%). This increase in residual velocity shows that, as the fiber content increases, there is a reduction in the material’s ability to dissipate kinetic energy, resulting in lower impact absorption efficiency and greater residual energy transmitted to the projectile. In practical terms, this means that the ER10 composite performs better in decelerating the projectile, while the ER30, although structurally more rigid, dissipates less energy during impact.

From a microstructural point of view, the observed behavior is associated with changes in the balance between matrix and reinforcement. In the ER10 composite, the smaller volume of fibers favors a more uniform impregnation by the epoxy matrix, ensuring good interfacial adhesion and greater participation of the matrix in dissipative mechanisms [2], such as microcracking, delamination, and plastic deformation. This condition provides a more gradual failure regime, allowing the material to absorb a greater amount of energy before complete rupture. As the fiber fraction increases, as in ER20 and ER30, impregnation becomes less efficient, and microvoids and discontinuities appear [14,15], hindering the transfer of stresses between the matrix and reinforcement [16]. The material then exhibits more brittle behavior and less efficient energy absorption, even while maintaining its physical integrity after impact.

The efficiency of stress transfer in fiber-reinforced composites is strongly governed by the quality of the fiber–matrix interface. Adequate interfacial adhesion enables effective load transfer from the matrix to the reinforcing fibers, thereby improving the mechanical response and energy dissipation capacity of the composite. Conversely, insufficient adhesion or incomplete matrix impregnation may reduce the efficiency of stress transfer and promote premature interfacial debonding during loading. Previous studies have systematically demonstrated that fiber–matrix adhesion plays a critical role in determining the mechanical strength of epoxy-based fiber composites, with a clear correlation between interfacial bonding quality and tensile performance. In particular, it has been shown that adhesion and impregnation efficiency can independently influence the strength of fiber-reinforced polymer systems, highlighting the importance of both mechanisms in controlling composite performance [17,18].

Another relevant point is the reduction in the standard deviation of residual velocities with increasing fiber content: 2.48 m/s for ER10, 2.12 m/s for ER20, and 1.57 m/s for ER30. This decrease in dispersion indicates that, although the energy performance of ER30 is lower, its impact behavior is more predictable and uniform. This characteristic can be advantageous in multilayer configurations, where structural consistency and penetration resistance are desired.

### 3.2. Energy-Based Ballistic Parameters

The equivalent ballistic limit velocity (V_L,eq_) and absorbed energy (E_abs_) values calculated from the experimental data are presented in Table 3. A clear decreasing trend is observed in both parameters as the raffia content increases from 10 to 30 vol.%.

The results presented for the equivalent ballistic limit (V_L,eq_) and absorbed energy (E_abs_) of epoxy composites reinforced with raffia fabric clearly indicate the influence of the fiber volume fraction on the ballistic performance of the materials. A progressive reduction is observed in both the equivalent ballistic limit and the absorbed energy as the fiber content in the epoxy matrix increases. The ER10 composite (10% raffia) showed the best results, with an average V_L,eq_ of 210.14 ± 5.81 m/s and absorbed energy of 176.74 ± 9.71 J, demonstrating greater efficiency in dissipating the projectile’s kinetic energy. ER20 (20%) obtained intermediate values—V_L,eq_ of 172.95 ± 1.93 m/s and E_abs_ of 119.66 ± 2.67 J— while ER30 (30%) showed the worst performance, with an average V_L,eq_ of 139.17 ± 7.90 m/s and absorbed energy of only 77.67 ± 8.96 J. This represents reductions of approximately 34% and 56%, respectively, compared to the composite with the lowest fiber fraction.

These results show that increasing the fiber content significantly reduces the energy absorption capacity and deceleration efficiency of the projectile. The explanation for this behavior is associated with the material’s microstructure and the quality of the fiber/matrix interface. In the ER10 composite, the lower fiber volume favors more uniform impregnation and better interfacial adhesion, allowing for more efficient stress transfer and the development of dissipative mechanisms, such as matrix microcracking, delamination, and partial fiber pull-out. These mechanisms consume energy gradually and in a controlled manner, resulting in a tougher and more efficient response during impact.

With the increase in the fiber fraction in ER20, and especially in ER30, the matrix begins to play a secondary role in energy dissipation. It is plausible that higher fiber packing may have reduced effective matrix impregnation, potentially limiting interfacial stress transfer. In this way, the material becomes more rigid and brittle, exhibiting abrupt failures and a reduced capacity to dissipate energy before projectile penetration. This behavior may be associated with reduced impregnation efficiency at higher fiber volume fractions, which can limit the quality of the fiber–matrix interface and consequently reduce the effectiveness of stress transfer mechanisms. Similar relationships between fiber–matrix adhesion, impregnation quality, and composite mechanical performance have been reported for epoxy-based fiber composites in previous studies [17,18].

Analysis of the variability of the results reinforces these observations. The standard deviation of V_L,eq_ and E_abs_ increases for ER30, indicating more inconsistent behavior and sensitivity to microstructural imperfections. In contrast, ER20 showed the lowest standard deviation, suggesting more reproducible and stable performance. This indicates that, although ER30 is the least efficient in terms of energy absorption, it exhibits superior stiffness and more predictable behavior, characteristics that can be advantageous in structural applications or in multilayer shielding systems, where different materials act synergistically.

The mass-normalized ballistic performance of the composites is summarized in Table 4, which presents the absorbed energy (E_abs_), target mass, and specific energy absorption (SEA) for each configuration.

In addition to the absolute absorbed energy, a mass-normalized analysis was performed through the specific energy absorption (SEA), defined as the ratio between the absorbed energy and the target mass. The SEA results are presented in Table 4.

The obtained values were 899.60 J/kg for ER10, 619.58 J/kg for ER20, and 409.23 J/kg for ER30, confirming the same decreasing trend observed for the total absorbed energy. This indicates that the reduction in ballistic performance with increasing raffia content is not only a consequence of increased mass, but is intrinsically related to the reduced efficiency of energy dissipation mechanisms.

Therefore, even when evaluated on a mass basis, the ER10 configuration remains the most efficient in absorbing impact energy, reinforcing that lower fiber volume fractions provide a more favorable balance between matrix continuity, interfacial interaction, and damage evolution under high-velocity impact.

A broader analysis of energy-based and dynamic parameters is provided in Table 5, which summarizes the absorption efficiency, residual energy, and momentum reduction for each laminate configuration.

The results solidify a consistent picture: the higher the raffia content, the lower the panel’s ability to dissipate impact energy and “brake” the projectile. Absorption efficiency drops from 23.48% (ER10) to 15.95% (ER20) and 10.30% (ER30), approximate reductions of 32% and 56% compared to ER10. In parallel, the residual energy transmitted to the projectile increases from 575.6 J to 630.6 J and 676.1 J (≈+10% and +17.5% vs. ER10), showing that more energy “passes” through the panels as the fiber fraction increases. The dynamic metric reaches the same conclusion: the reduction in linear momentum decreases from 0.1253 to 0.0832 and 0.0529 (−33.6% and −57.8% vs. ER10), showing a progressive loss of braking power.

The reduction in linear momentum (Δ*p*) provides additional insight into the interaction between the projectile and the target, particularly in terms of impulse transmission and deformation mechanisms. Physically, Δ*p* represents the portion of the projectile’s momentum that is transferred to the composite during impact, being directly associated with the forces developed at the projectile–target interface and the duration of the interaction.

Higher Δ*p* values, as observed for the ER10 configuration, indicate a more effective transfer of momentum to the target, which is typically associated with greater deformation, a greater reduction in projectile velocity, and more extensive activation of energy dissipation mechanisms such as matrix cracking, fiber pull-out, and interfacial friction. These mechanisms contribute to a more efficient “braking” of the projectile.

In contrast, the lower Δ*p* values obtained for ER20 and ER30 suggest a reduced ability to transmit impulse and deform the structure, resulting in a shorter reduction in projectile velocity and a more ballistic-type perforation with less resistance to penetration. This behavior is consistent with the higher residual velocities and lower absorbed energies observed for these configurations, reinforcing the interpretation that increasing the raffia content reduces the overall efficiency of impact energy dissipation.

The absorbed energy can be further interpreted in terms of the specific contribution of the raffia fibers to the overall dissipation mechanisms. In natural fiber-reinforced composites, energy absorption under high-velocity impact is governed by a combination of matrix deformation, fiber-related mechanisms, and interfacial interactions.

At lower fiber volume fractions, as in the ER10 configuration, the raffia fibers are more effectively embedded within the epoxy matrix, enabling efficient stress transfer and the activation of progressive energy dissipation mechanisms. These include fiber pull-out, interfacial debonding, and frictional sliding, which contribute significantly to energy absorption by dissipating energy over a larger reduction in projectile velocity and volume.

As the fiber content increases (ER20 and ER30), the effectiveness of these mechanisms tends to decrease. Higher fiber packing can lead to reduced matrix continuity, incomplete impregnation, and increased void content, which limit interfacial bonding and reduce the contribution of frictional and pull-out mechanisms. As a result, the energy dissipation becomes less efficient, leading to a more localized and brittle-like response under impact.

This behavior explains the observed reduction in absorbed energy with increasing raffia content, highlighting that the contribution of the fibers is not only dependent on their presence, but strongly governed by their distribution, interaction with the matrix, and the resulting damage evolution during impact.

Although the present study focuses on the macroscopic ballistic performance of the composites, it is important to discuss the underlying failure mechanisms typically associated with natural fiber-reinforced polymer systems under high-velocity impact. In such materials, energy dissipation is commonly governed by a combination of mechanisms, including fiber pull-out, matrix cracking, fiber breakage, and interfacial debonding, as widely reported in the literature.

For raffia-based composites, these mechanisms are expected to be strongly influenced by the lignocellulosic structure of the fibers, their surface roughness, and the fiber–matrix adhesion quality, which can promote frictional energy dissipation and progressive damage evolution during impact.

However, a detailed microscopic investigation, such as scanning electron microscopy (SEM), was not included in the present work, as the primary objective was to evaluate the ballistic performance at the structural level. Future studies are planned to incorporate fractographic analyses in order to correlate the observed macroscopic response with the underlying damage mechanisms at the microstructural scale.

The fiber–matrix interfacial adhesion plays a critical role in the mechanical performance of natural fiber-reinforced composites, particularly under dynamic loading conditions. In the present study, no surface treatment was applied to the raffia fibers, and no direct measurements of interfacial properties, such as interfacial shear strength, were performed.

However, the observed ballistic behavior provides indirect evidence of the effectiveness of the fiber–matrix interaction. The higher energy absorption efficiency observed for the ER10 configuration suggests that moderate fiber content may favor more effective stress transfer and energy dissipation mechanisms, such as frictional sliding and progressive fiber pull-out. In contrast, the reduction in performance at higher fiber volume fractions (ER20 and ER30) may be associated with reduced wetting, increased void content, and weaker interfacial bonding, which can limit load transfer efficiency and promote premature failure.

These findings are consistent with the general behavior reported in natural fiber composites, where the balance between fiber content and interfacial quality governs the overall mechanical response. Future studies should include surface treatments and direct characterization of interfacial properties to further elucidate the role of adhesion in the ballistic performance of raffia-reinforced systems.

It is also important to consider that natural fibers are inherently sensitive to moisture absorption and environmental conditions, which can significantly affect their mechanical performance and interfacial behavior. The hydrophilic nature of lignocellulosic fibers may lead to swelling, plasticization, and degradation of the fiber–matrix interface over time.

In the present study, the ballistics tests were conducted under controlled laboratory conditions, and no environmental aging or moisture conditioning was applied prior to testing. Therefore, the results reflect the intrinsic performance of the composites in their as-fabricated state.

It is expected that prolonged exposure to humid or aggressive environments may alter the ballistic response of raffia-reinforced composites, particularly by weakening interfacial adhesion and reducing energy dissipation capability. Future studies should address environmental conditioning effects to better assess the long-term performance and durability of these materials in real-world applications.

The variability of the data helps to interpret the mechanism. In terms of efficiency, the coefficient of variation (CV) is ~4.0% in ER10, falls to ~1.6% in ER20, and rises to ~10.9% in ER30; for moment reduction, the CVs are ~4.2%, ~1.7%, and ~11.2%, respectively. That is, ER20 exhibits more reproducible behavior (low dispersion), while ER30, in addition to being less energy-efficient, is more sensitive to small microstructural variations (voids, poorly impregnated regions, misalignments). Residual energy, being dominated by a relatively stable V_r_ (value close to 1.3%), shows low CVs in all cases (≤1.3%), reinforcing the reliability of the test.

From a micromechanical point of view, the superior performance of the ER10 is consistent with greater matrix participation and improved wettability/fiber–matrix interaction, activating dissipative mechanisms (matrix microcracking, progressive delamination, pull-out friction) that convert kinetic energy into distributed damage [14]. In the ER20, these mechanisms still operate, but with less intensity; the lower variability suggests a more stable architecture, possibly with fiber packing that does not severely compromise anchoring. In the ER30, the matrix becomes deficient in “stitching” and anchoring the bundles, favoring dry zones and abrupt delamination’s: the damage evolves in a less stable and less dissipative way, reducing efficiency and momentum reduction and leaving more residual energy in the projectile [15,19]. These observations are consistent with established interfacial mechanics concepts in fiber-reinforced composites, where the combined effects of fiber–matrix adhesion and impregnation quality govern the efficiency of stress transfer and the resulting mechanical response of the material [17,18].

It should be noted that the present study was limited to fiber volume fractions up to 30 vol.%, which is within the range commonly reported for hand lay-up processed natural fiber composites. At higher fiber contents, processing limitations such as reduced resin impregnation, increased void content, and impaired fiber–matrix interaction may become more pronounced, potentially compromising the structural integrity and reproducibility of the laminates.

Although additional fiber volume fractions could provide further insight into the structure–performance relationship, the results obtained in this work already establish a clear and consistent trend of decreasing energy absorption capability with increasing raffia content. Therefore, the selected range is considered sufficient to capture the dominant mechanisms governing the ballistic response under the processing conditions adopted.

Future investigations may extend this analysis to higher fiber volume fractions and alternative manufacturing techniques, such as vacuum-assisted processes, to evaluate whether improved impregnation conditions can modify the observed trends.

### 3.3. Projectile Velocity–Time Analysis

**Figure 4:**
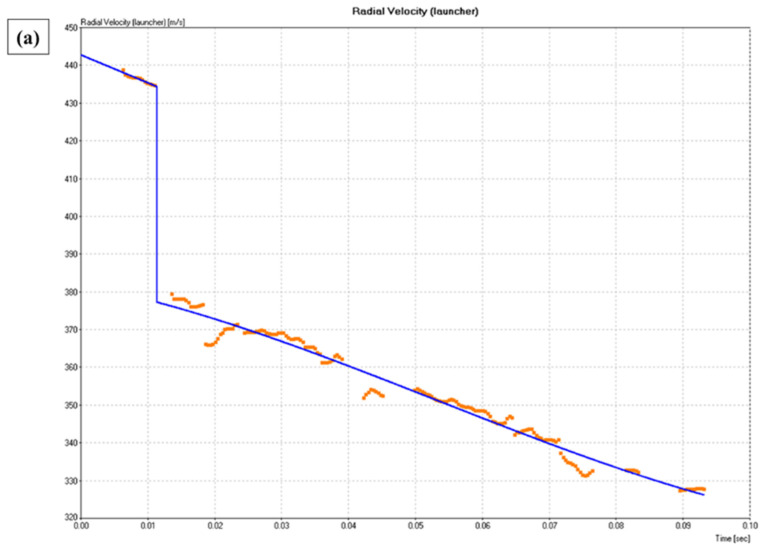

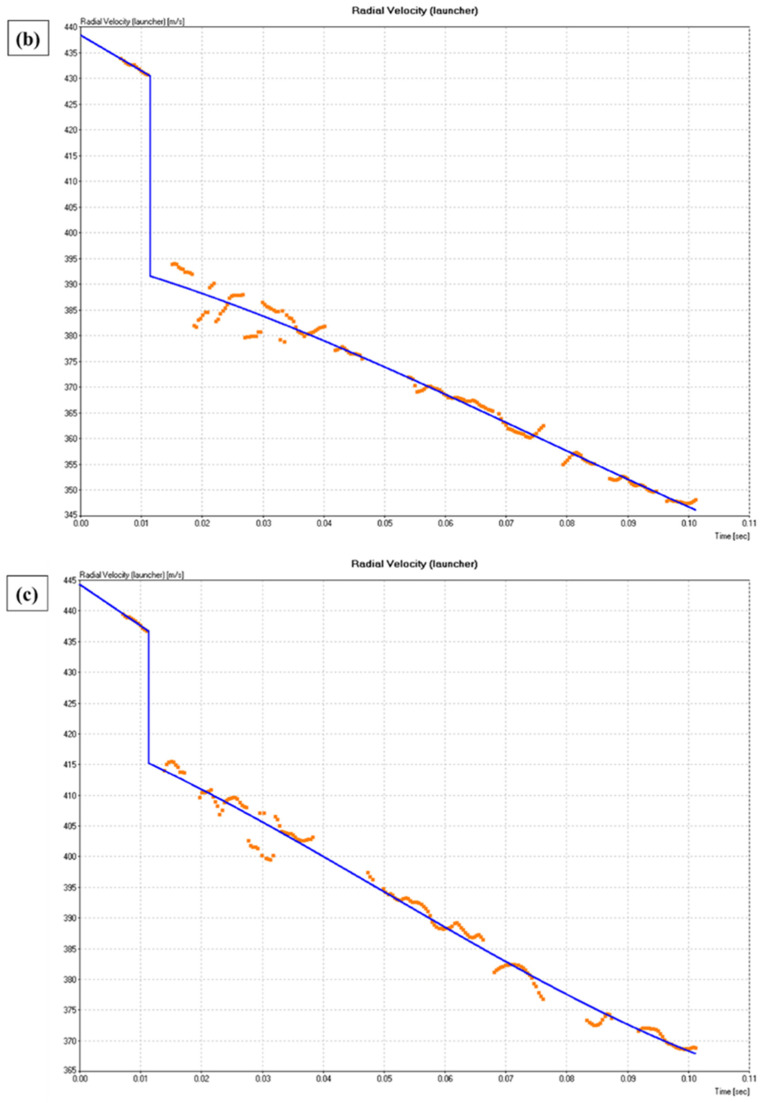
Temporal evolution of the longitudinal velocity of the projectile during the ballistic test with 9 mm ammunition for the epoxy composite reinforced with raffia fabric: (**a**) ER10 (10%), (**b**) ER20 (20%) and (**c**) ER30 (30%).

The projectile velocity–time histories recorded during the ballistics tests are shown in Figure 4.

Figure 4 shows the temporal evolution of the longitudinal velocity of the projectile during ballistic impact with 9 mm ammunition for epoxy composites reinforced with raffia fabric in volume fractions of 10%, 20% and 30% (ER10, ER20 and ER30). The combined analysis of the curves allows for a direct evaluation of the differences in the projectile deceleration process and, consequently, in the energy dissipation mechanisms activated in each configuration.

In all cases, an initial pre-impact regime is observed, characterized by an approximately constant velocity between 435 and 445 m/s, corresponding to the impact velocity (V_i_). The abrupt velocity drop observed around t ≈ 0.01 s represents the moment at which the projectile crosses the target plane within the radar acquisition window. It should be emphasized that the actual magnitude of projectile velocity reduction is on the order of tens of microseconds, considering the laminate thickness (~12 mm) and impact velocity (~430 m/s), and is therefore not temporally resolved by the Doppler radar system.

The magnitude of this drop varies significantly between configurations. In the ER10 composite (10%), the instantaneous velocity reduction is the most pronounced, with a difference on the order of 54 m/s between the velocities immediately before and after impact. This behavior indicates a relatively longer magnitude of projectile velocity reduction and the activation of more efficient dissipative mechanisms, such as progressive matrix fracture, interlaminar delamination, and controlled fiber pull-out. As a result, the projectile emerges from the panel with lower residual velocity, consistent with the average V_r_ values and the highest absorbed energy observed experimentally for this configuration.

For the ER20 composite (20%), the initial velocity drop is intermediate, demonstrating a moderate projectile deceleration capacity. The slope of the curve immediately after impact is less pronounced than in the ER10, reflecting less energy dissipation during penetration. However, the curve shows less dispersion and fluctuation over time, indicating more stable projectile behavior after passing through the target. This characteristic suggests a more homogeneous structural response of the panel, consistent with the lower standard deviations observed in the energy and efficiency metrics for the ER20.

In the case of the ER30 composite (30%), the instantaneous velocity reduction is clearly the least pronounced among the three configurations. The projectile maintains a significantly higher residual velocity immediately after penetration, indicating that a smaller fraction of the incident kinetic energy was transferred to the material. This behavior is consistent with the greater stiffness of the laminate and the lower efficiency of dissipative mechanisms, resulting in a “cleaner” penetration and a shorter effective magnitude of projectile velocity reduction. Under high strain-rate perforation, excessive stiffness may reduce the duration of mechanical coupling between projectile and target, limiting the activation of progressive damage mechanisms such as matrix cracking, delamination, and fiber pull-out. Consequently, ER30 exhibits the lowest values of absorbed energy, absorption efficiency, and momentum reduction.

After the impact phase, all curves exhibit an approximately linear deceleration regime, predominantly associated with aerodynamic drag during the projectile’s flight to the remote sensor. The inclination differences in this section are relatively small, indicating that the post-impact behavior is mainly governed by flight conditions and not by additional interactions with the target. However, the fluctuations observed in the experimental signals (especially in the discrete data) tend to be more pronounced in the ER10 and ER30 cases, and may be related to small variations in projectile orientation (yaw effect), partial fragmentation, or instrumental noise from the Doppler system. These oscillations do not compromise the overall analysis but reinforce the importance of interpreting the residual velocity as an average value obtained in a region close to the target’s exit plane.

From a comparative point of view, the joint analysis of the curves clearly shows the trade-off between energy dissipation capacity and structural stiffness. ER10 maximizes projectile deceleration and energy dissipation, making it more suitable for absorption functions in frontal or sacrificial layers. ER20 exhibits intermediate behavior, with lower energy efficiency but greater stability and reproducibility, desirable characteristics for transition layers. ER30, while preserving greater structural integrity after impact, demonstrates limited braking and energy dissipation capacity, making it less suitable for dissipative functions, but potentially adequate as a structural support layer in multilayer systems.

### 3.4. Statistical Validation of Ballistic Parameters

The statistical results of the one-way ANOVA applied to absorbed energy (E_abs_) are presented in Table 6.

The analysis of variance (ANOVA) of the absorbed energy of epoxy composites reinforced with raffia fabric in volume fractions of 10%, 20%, and 30% shows, in a statistically robust way, that the fiber content exerts a significant influence on the impact performance of these materials. The high value of the F statistic (204.7), associated with a *p*-value less than 0.001, allows us to reject with a high degree of confidence the null hypothesis of equality between the means, indicating that the differences observed between the three groups are not random, but result directly from the variation in the reinforcement content.

The decomposition of the total variability reinforces this interpretation, since the sum of squares between groups represents approximately 97% of the total sum of squares, while only about 3% of the variability is associated with differences within each group. This result demonstrates that most of the variation in absorbed energy is explained exclusively by the raffia content, highlighting both the strong sensitivity of the analyzed property to the factor under study and the low experimental dispersion. The reduced intragroup variability indicates good repeatability of the tests and adequate control of the composite manufacturing process, conferring high reliability to the average values obtained.

From a physical–mechanical point of view, the statistical results are consistent with the expected behavior for polymer composites reinforced with natural fibers. At lower reinforcement contents, such as in ER10, the epoxy matrix still plays a predominant role, allowing for higher levels of deformation and activating efficient energy dissipation mechanisms, such as matrix microcracking, fiber pull-out, and interfacial friction [8,9,10]. As the raffia content increases to 20% and 30%, there is an increase in material stiffness and greater restriction of overall deformation, which significantly alters the energy absorption mechanisms, favoring phenomena such as delamination and more localized fracture, with a direct impact on the total energy dissipated.

The high F-value indicates that this change in behavior is not gradual or marginal, but rather significant, reflecting a clear transition in the impact response mode as a function of fiber volume fraction. Although the ANOVA unequivocally confirms the existence of overall differences between the groups, it does not identify which configurations differ from each other. Given the extreme level of significance observed, the application of post hoc tests, such as Tukey’s test, becomes statistically justifiable and methodologically recommended in order to discriminate the specific differences between the pairs of composites and establish a clear hierarchy of energy performance.

The pairwise comparisons obtained through Tukey’s HSD test are summarized in Table 7.

The Tukey multiple comparisons test applied to the absorbed energy (E_abs_) results of epoxy composites reinforced with raffia fabric in volume fractions of 10%, 20%, and 30% decisively complements the overall analysis provided by ANOVA, allowing for the clear and unequivocal identification of which configurations differ statistically from each other. The results indicate that all pairwise comparisons are statistically significant, since, in all cases, the observed mean differences far exceed the critical value of HSD (13.10 J).

The comparison between ER10 and ER20 shows an average difference of 57.08 J, a value more than four times higher than the critical limit established by the test. This result demonstrates that increasing the raffia content from 10% to 20% causes a substantial change in the composite’s energy absorption capacity, confirming that this transition represents a real and consistent change in impact behavior, and not just an experimental fluctuation. From a mechanical point of view, this effect can be associated with the modification of energy dissipation mechanisms, with a reduction in matrix deformability and a greater influence of the fibrous reinforcement in controlling the structural response.

The difference becomes even more pronounced when comparing ER10 and ER30, where the average difference reaches 99.08 J, almost eight times higher than the critical HSD. This result demonstrates that increasing the fiber content to 30% leads to an even more intense transformation in the material’s energy behavior. Statistically, this is a very clear separation between the groups, indicating that the composite with the lower fiber fraction exhibits a completely different energy absorption regime from that observed at high reinforcement contents. Physically, this behavior can be attributed to the significant increase in stiffness and the greater restriction of the overall deformation of the laminate, which limits the more efficient dissipative mechanisms associated with the polymer matrix.

Even the comparison between ER20 and ER30, whose average difference is the smallest among the pairs analyzed (41.99 J), remains significantly higher than Tukey’s critical value. This result indicates that, although both composites have high reinforcement contents, there is still a statistically significant difference between their impact behaviors. This suggests that the mechanical response does not fully stabilize from 20% raffia onwards, but remains sensitive to increases in volume fraction, demonstrating that small variations in fiber content, at higher regimes, still produce measurable and relevant effects on absorbed energy.

From a methodological point of view, the fact that all comparisons showed statistical significance reinforces the robustness of the experimental results and confirms the high magnitude of the effect of the “fiber content” factor already evidenced by the ANOVA. Furthermore, the significant distance between the mean differences and the critical HSD indicates a large effect size, dispelling any doubt about the practical relevance of the observed differences. This is not merely a matter of statistical significance but of differences with a direct impact on materials engineering.

The ANOVA results for energy absorption efficiency are presented in Table 8.

The analysis of variance (ANOVA) of the absorption efficiency of epoxy composites reinforced with raffia fabric in volume fractions of 10%, 20%, and 30% clearly and statistically robustly demonstrates that the reinforcement content exerts a decisive influence on this property. The high value of the F statistic = 299.5, associated with a *p*-value less than 0.001, allows us to reject with a very high level of confidence the null hypothesis of equality between the means, confirming that the differences observed between the groups are real and directly associated with the variation in fiber content.

The decomposition of the total variability strongly reinforces this conclusion. The sum of squares between the groups (436.2) represents approximately 98.0% of the total sum of squares (444.9), while only about 2.0% of the variability is attributed to fluctuations within the groups. This result indicates that absorption efficiency is strongly controlled by the volume fraction of raffia, with minimal contribution from intrinsic experimental variability. In practical terms, this demonstrates high repeatability of the tests, good control of the composite manufacturing process, and homogeneity of the samples within each configuration.

From a statistical point of view, the mean square between groups (MS = 218.1) is almost 300 times greater than the mean square within groups (MS = 0.73), highlighting an extremely marked contrast between the analyzed configurations. This relationship explains the exceptionally high value of the F statistic and indicates a very large effect size, which rules out any interpretation of purely statistical significance. It is clearly an effect with substantial physical and engineering relevance.

From a physical–mechanical perspective, these results are directly related to the energy dissipation mechanisms during impact. Absorption efficiency, considering the ratio between absorbed and incident energy, is particularly sensitive to the material’s ability to convert kinetic energy into internal dissipative mechanisms. In composites with lower fiber content, such as ER10, the higher proportion of the epoxy matrix favors more extensive deformations and the activation of mechanisms such as matrix microcracking, fiber pull-out, and interfacial friction, resulting in greater absorption efficiency [10,11,12]. As the raffia content increases to 20% and 30%, the laminate stiffness increases and the overall deformability is progressively restricted, reducing the fraction of incident energy effectively dissipated, which is directly reflected in the decrease in absorption efficiency.

The high F-value indicates that this behavioral transition is abrupt and well-defined, characterizing clear changes in the impact response regime as the reinforcement level increases. Similar to the analysis of absorbed energy, the ANOVA does not specify which pairs of groups differ from each other, but the extreme level of statistical significance observed makes the application of post hoc tests, such as Tukey’s test, not only justifiable but essential to discriminate the specific differences between ER10, ER20, and ER30 and establish a precise performance hierarchy in terms of efficiency.

The Tukey multiple comparison results for absorption efficiency are shown in Table 9.

Tukey’s multiple comparisons test applied to the absorption efficiency results of epoxy composites reinforced with raffia fabric at volume fractions of 10%, 20%, and 30% provides a clear and statistically unequivocal discrimination between all configurations analyzed. In all pairwise comparisons, the observed mean differences far exceed the critical HSD value (1.43%), confirming that the three composites exhibit distinct behaviors regarding the efficiency with which they convert incident energy into effectively dissipated energy.

The comparison between ER10 and ER20 shows an average difference of 7.53%, a value more than five times higher than the critical HSD. This result demonstrates that increasing the raffia content from 10% to 20% causes a significant and statistically robust reduction in absorption efficiency. From a mechanical point of view, this difference indicates that increasing the fiber fraction is already sufficient to significantly alter the predominant dissipative mechanisms, reducing the contribution of the epoxy matrix, which is responsible for a large part of the energy dissipation through deformation and microcracking.

The difference becomes even more pronounced when comparing ER10 and ER30, where the average difference reaches 13.18%, almost ten times higher than Tukey’s critical value. This result categorically demonstrates that the composite with a higher raffia content exhibits a clearly distinct impact response regime from that with a lower reinforcement fraction. Statistically, this is a very well-defined separation between the groups, while, from a physical point of view, it reflects the significant increase in laminate stiffness and the consequent limitation of more efficient energy dissipation mechanisms, resulting in a smaller fraction of incident energy effectively absorbed.

Even the comparison between ER20 and ER30, which shows the smallest average difference (5.65%), remains significantly higher than the critical HSD, confirming that, even at higher reinforcement contents, absorption efficiency remains sensitive to variations in the fiber volume fraction. This result indicates that there is no saturation of the raffia content effect from 20% onwards, but rather a continuous trend of modification of the energy behavior as the reinforcement content increases.

From a methodological point of view, the fact that all comparisons showed statistical significance reinforces the robustness of the experimental data and fully validates the conclusions obtained from the ANOVA. The observed differences not only have statistical significance but also high practical relevance, since percentage variations of this magnitude directly impact the efficiency of impact absorption and ballistic protection systems.

### 3.5. Post-Impact Damage Patterns

**Figure 5:**
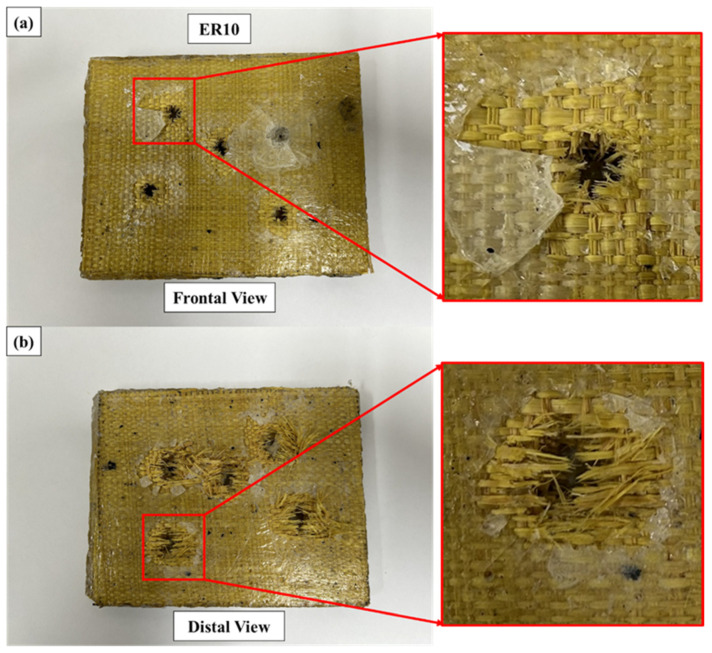
Macroscopic post-impact damage patterns of the ER10 laminate (10 vol.% raffia fabric) subjected to 9 mm full metal jacket projectile impact: (**a**) impact (front) face and (**b**) rear (exit) face.

Representative macroscopic damage patterns observed in the ER10 laminate after ballistic impact are presented in Figure 5.

The ER10 laminate exhibited the most extensive macroscopic damage among the investigated configurations. On the impact face, the perforation openings displayed irregular geometry accompanied by pronounced radial matrix cracking and localized fragmentation extending beyond the nominal projectile diameter. This suggests progressive material failure rather than abrupt perforation.

On the rear face, significant fiber pull-out and fibrillar expansion were observed around the exit region. The damage area was visibly larger than the projectile diameter, indicating that energy dissipation mechanisms such as matrix microcracking, interfacial friction, and progressive delamination were actively engaged during projectile penetration.

Such damage morphology suggests that the dominant failure mechanisms involved matrix-dominated cracking combined with extensive interlaminar shear and tensile fiber rupture, promoting progressive energy dissipation rather than localized shear plugging [14,19,20]. The visibly expanded damage area beyond the nominal projectile diameter indicates significant in-plane stress redistribution and prolonged projectile–target interaction.

The extensive damage morphology is consistent with the highest absorbed energy (176.74 ± 9.71 J), absorption efficiency (23.48 ± 0.93%), and momentum reduction (0.1253 ± 0.0053) measured for ER10. The macroscopic observations support the interpretation that the lower fiber volume fraction promotes improved matrix continuity and interfacial interaction, enabling distributed and progressive energy dissipation during ballistic impact.

**Figure 6:**
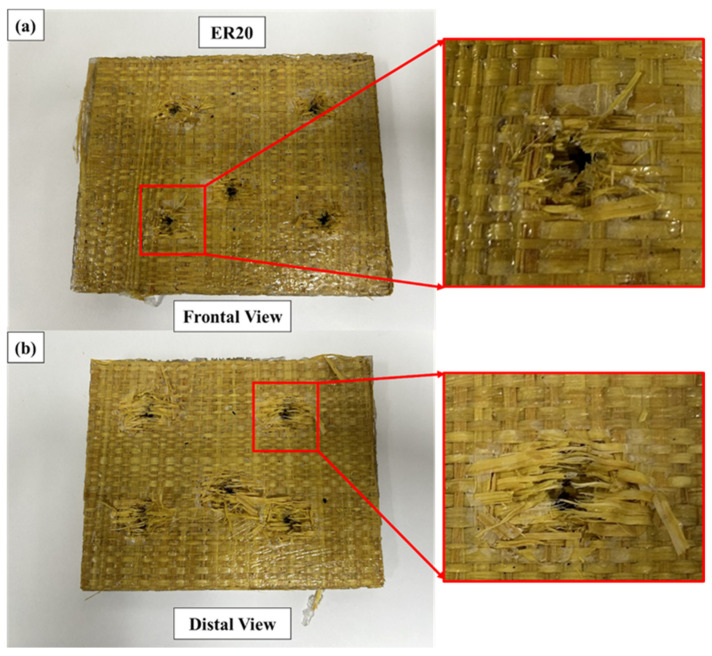
Macroscopic post-impact damage patterns of the ER20 laminate (20 vol.% raffia fabric) subjected to 9 mm full metal jacket projectile impact: (**a**) impact (front) face and (**b**) rear (exit) face.

Representative macroscopic damage patterns observed in the ER20 laminate after ballistic impact are presented in Figure 6.

Compared to ER10, the perforation morphology exhibits a more defined and geometrically confined hole, with reduced radial matrix cracking and less extensive surface fragmentation. The damage zone remains visible but is more localized around the projectile trajectory.

On the rear face, moderate fiber pull-out and limited fibrillar expansion are observed. Although progressive damage mechanisms are still active, the extension of delamination and matrix cracking appears less pronounced than in ER10. This morphological transition indicates a reduced contribution of matrix-dominated dissipation mechanisms and a shorter magnitude of projectile velocity reduction [21].

In this configuration (ER20), the failure process appears to transition toward a more balanced contribution between matrix cracking and fiber-dominated tensile rupture, with reduced interlaminar shear propagation compared to ER10. This shift suggests a partial restriction of progressive delamination mechanisms due to increased laminate stiffness [19].

The observed damage patterns are consistent with the intermediate absorbed energy (119.66 ± 2.67 J), absorption efficiency (15.95 ± 0.26%), and momentum reduction (0.0832 ± 0.0014), confirming that increasing the fiber volume fraction alters the balance between stiffness and energy dissipation without completely suppressing progressive failure mechanisms.

**Figure 7:**
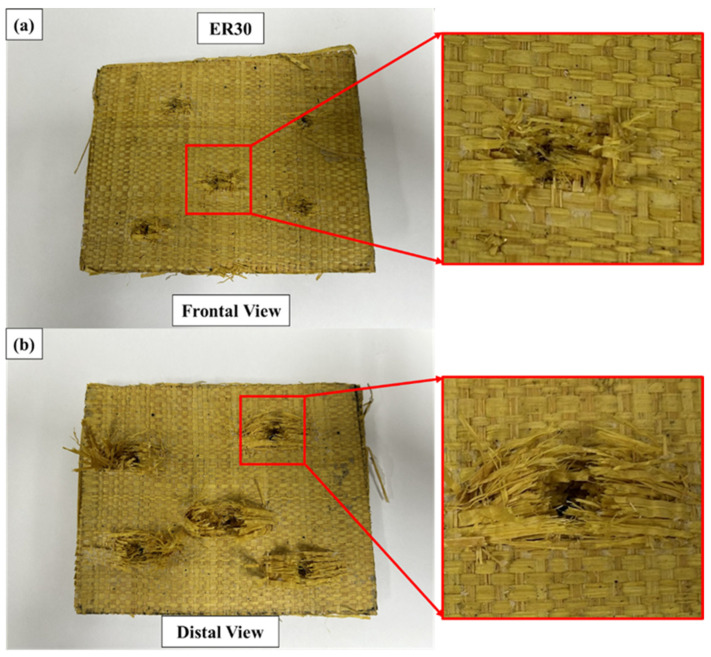
Macroscopic post-impact damage patterns of the ER30 laminate (30 vol.% raffia fabric) subjected to 9 mm full metal jacket projectile impact: (**a**) impact (front) face and (**b**) rear (exit) face.

Representative macroscopic damage patterns observed in the ER30 laminate after ballistic impact are presented in Figure 7.

On the impact face, the perforation openings appeared more geometrically defined, with reduced radial cracking and limited matrix fragmentation compared to ER10 and ER20. The damage zone was largely restricted to the immediate vicinity of the projectile trajectory, indicating a more abrupt penetration process.

On the rear face, fiber pull-out and fibrillar expansion were visibly less pronounced, and the exit openings appeared comparatively compact. The reduced extension of delamination and matrix cracking suggests a shorter magnitude of projectile velocity reduction and diminished activation of progressive dissipative mechanisms.

The failure mode in ER30 appears to approach a more localized penetration mechanism, possibly dominated by shear plugging and limited tensile fiber rupture, consistent with a stiffer but less energy-dissipative response [20,22]. The confined damage footprint further indicates restricted stress redistribution within the laminate.

These observations are consistent with the lowest absorbed energy (77.67 ± 8.96 J), absorption efficiency (10.30 ± 1.12%), and momentum reduction (0.0529 ± 0.0059) measured for ER30. The higher fiber packing likely increased laminate stiffness while limiting matrix continuity and interfacial energy dissipation, resulting in a more localized and less energy-efficient perforation mode.

Although no scanning electron microscopy (SEM) analysis was performed, the macroscopic damage patterns provide consistent qualitative evidence supporting the energy-based and statistical findings discussed previously. The progressive transition from distributed damage (ER10) to increasingly localized penetration (ER30) reinforces the interpretation that laminate stiffness and matrix continuity govern the balance between energy dissipation and structural integrity under handgun-level ballistic impact.

### 3.6. Comparative Analysis with Literature

A comparative overview of the present results and selected literature data is provided in Table 10.

Based on the values presented in Table 9, it is possible to perform a robust critical analysis of the ballistic performance of epoxy composites reinforced with raffia fabric in relation to other systems evaluated under impact from 9 mm projectiles, provided that the actual severity level of the test, the target architecture, and the dominant physical mechanisms in each material are carefully considered.

In addition to the natural fiber-based systems discussed above, it is important to contextualize the present results with respect to conventional high-performance ballistic materials, such as aramid fibers (e.g., Kevlar™), ultra-high-molecular-weight polyethylene (UHMWPE), and glass fiber-reinforced polymers (GFRP). These materials are widely used in ballistic protection systems due to their high strength, high toughness, and well-established energy absorption mechanisms.

Compared to these synthetic systems, the raffia-reinforced epoxy composites investigated in this work exhibit lower absolute ballistic performance in terms of energy absorption and penetration resistance, which is expected given the intrinsic mechanical limitations of natural lignocellulosic fibers. However, it is important to emphasize that the objective of natural fiber composites is not to directly replace high-performance materials, but rather to act as complementary components in multilayered armor systems (MAS), where different layers perform distinct mechanical functions.

In this context, the energy absorption efficiency observed for the ER10 configuration (23.48%) under relatively high impact energy (~750 J) demonstrates that raffia-based composites can provide meaningful energy dissipation capacity when used in combination with harder strike faces or high-performance backing materials. Furthermore, their lower density, reduced cost, and improved sustainability make them attractive candidates for intermediate or backing layers in hybrid ballistic structures.

Although all studies use 9 mm ammunition, the results make it clear that this does not imply equivalent impact conditions. In the present work, the average impact velocities are around 433–434 m/s, which, for a projectile mass of 8 g, corresponds to an incident energy on the order of 750 J. In contrast, the study of the veneer–aramid hybrid works with velocities close to 335 m/s, resulting in a significantly lower initial energy, around 450 J, while the HDPE/rattan system operates in an even less severe regime, with initial energies between 410 and 420 J. This difference is fundamental, as it implies that the raffia/epoxy composites were subjected to a considerably more energetic impact, which must be taken into account when interpreting absolute values of absorbed energy.

From this perspective, absorption efficiency (%E_abs_) emerges as the most suitable comparative parameter. In the case of raffia composites, a clear and monotonic decrease in efficiency is observed with increasing fiber volume fraction: 23.48% for ER10, 15.95% for ER20, and 10.30% for ER30. This trend shows that, for the manufacturing process adopted, an excessive increase in fiber content compromises the material’s ability to dissipate energy, probably due to reduced effective matrix impregnation and weakened fiber–matrix adhesion. ER10, with a higher proportion of the epoxy matrix, favors progressive dissipative mechanisms—such as matrix microcracking, delamination, and controlled pull-out—which increase the magnitude of projectile velocity reduction and result in greater conversion of kinetic energy into internal damage.

When compared to the rattan fiber-reinforced HDPE system, the results show that ER10 and ER20 exhibit superior energy performance, even when operating under more severe impact conditions. The HDPE/rattan system achieves a maximum efficiency of only 13.68%, with an absorbed energy of approximately 57.6 J, characterizing a behavior dominated by perforation, with little projectile deceleration. This suggests that, despite the ductility of HDPE, the composite architecture and fiber–matrix interaction are not sufficient to activate effective dissipation mechanisms under rapid ballistic impact. In this context, the performance of ER10 is particularly relevant, as it demonstrates that a natural fiber-reinforced epoxy composite can outperform natural thermoplastic systems in terms of ballistic efficiency, provided there is an adequate balance between matrix and reinforcement.

It is important to note that direct comparisons of energy absorption efficiency (%E_abs_) between different studies must be interpreted with caution when impact velocities differ significantly. The ballistic response of fiber-reinforced polymer composites is known to be rate-dependent, and the mechanisms governing energy dissipation—such as fiber rupture, matrix cracking, delamination, and frictional pull-out—may vary with impact velocity. Consequently, the absorption efficiency of a given material may change as the impact energy increases. In the present study, the raffia-reinforced composites were tested at impact velocities around 433 m/s (≈750 J), whereas the veneer/aramid hybrid laminate (VAV2) reported in the literature was evaluated at approximately 335 m/s (≈451 J). Therefore, the efficiency values reported in Table 10 should be interpreted as indicative comparisons rather than strictly equivalent performance metrics. A fully rigorous comparison would require normalization procedures or testing under identical impact conditions.

The veneer/aramid hybrid laminate (VAV2) reported in the literature exhibits a high absorption efficiency of approximately 78.6%, corresponding to an absorbed energy of 354.7 J from an incident energy of about 451 J. However, it should be noted that this system was tested under a lower impact energy compared with the present study. In contrast, the ER10 composite evaluated in this work was subjected to a higher impact energy of approximately 750 J and absorbed about 176.7 J. Therefore, although the percentage efficiency of the VAV2 laminate appears higher, direct comparisons between efficiency values obtained under different threat levels must be interpreted with caution, since both the absolute energy levels and the impact conditions differ significantly. In this context, the results reported in Table 8 should be interpreted as indicative comparisons rather than direct performance equivalence between the different composite systems.

In addition to their mechanical performance, the use of raffia fibers as reinforcement also offers important sustainability advantages compared to conventional synthetic fibers. Natural lignocellulosic fibers are renewable, biodegradable, and generally associated with lower energy consumption during production, resulting in a reduced environmental footprint. Furthermore, raffia fibers are widely available in tropical regions, and their use can contribute to local economic development and value-added utilization of natural resources. Although no quantitative environmental impact assessment was performed in this study, the adoption of natural fiber-reinforced composites represents a promising pathway toward more sustainable material solutions in engineering applications. Therefore, the combination of acceptable ballistic performance with environmental and socio-economic benefits reinforces the potential of raffia-based composites as alternative materials, particularly in applications where sustainability is an important design criterion.

## 4. Conclusions

This study systematically evaluated the ballistic response of epoxy composites reinforced with raffia fabric at fiber volume fractions of 10, 20, and 30 vol.% under 9 mm full metal jacket projectile impact, maintaining equivalent impact velocities (~433 m/s) to ensure consistent comparison among configurations.

The results clearly demonstrate that increasing the raffia fiber content does not enhance ballistic energy dissipation under the manufacturing conditions adopted. On the contrary, the ER10 laminate (10 vol.% raffia) exhibited the highest absorbed energy (176.74 ± 9.71 J), the greatest absorption efficiency (23.48 ± 0.93%), and the largest momentum reduction (0.1253 ± 0.0053). Statistical analysis confirmed that the influence of fiber content on both absorbed energy and efficiency is highly significant (*p* < 0.001), with all pairwise comparisons validated by Tukey’s test. These findings establish a clear and statistically robust performance hierarchy among the investigated configurations.

The progressive reduction in ballistic efficiency observed for ER20 and ER30 indicates that excessive fiber volume fraction may compromise the material’s ability to dissipate kinetic energy during perforation. Under the present processing route, lower fiber content favors a more balanced interaction between matrix continuity and reinforcement contribution, resulting in superior deceleration capacity. In contrast, higher raffia fractions led to reduced energy absorption and increased residual velocity, despite maintaining structural integrity after impact.

Macroscopic post-impact damage patterns were consistent with the energy-based results, qualitatively corroborating the observed differences in projectile deceleration and penetration resistance. Although no microscopic (SEM) fractographic analysis was performed in this study, the combination of kinematic measurements, energy calculations, and rigorous statistical validation provides sufficient evidence to support the proposed interpretation of the ballistic response.

From an application-oriented perspective, the findings indicate that ER10 is the most suitable configuration for energy-dissipating roles, such as frontal or sacrificial layers in multilayered armor systems. ER20 presents intermediate behavior with low experimental dispersion, suggesting potential use as a transition layer in graded architectures. ER30, while less efficient in energy absorption, exhibits higher rigidity and post-impact integrity, which may be advantageous for structural backing functions.

The main contribution of this work lies in the quantitative demonstration—supported by statistical rigor—that lower raffia fiber fractions outperform higher fractions in terms of ballistic energy dissipation under handgun-level impact. By integrating energy-based metrics, momentum analysis, velocity–time history, and statistical validation under identical impact conditions, this study advances the rational design of sustainable natural fiber–reinforced composites for ballistic protection.

Future investigations incorporating detailed microscopic damage characterization and interfacial analysis are recommended to further elucidate the failure mechanisms governing the observed macroscopic ballistic behavior. Furthermore, the implementation of advanced numerical models based on finite element analysis (FEA), using tools such as LS-DYNA or ABAQUS, is proposed to provide deeper insight into stress wave propagation, damage evolution, and the influence of material architecture under high-velocity impact conditions.

## Figures and Tables

**Table 1:** Verification of ANOVA assumptions for ballistic parameters.

Variable	Shapiro–Wilk W	*p*-Value	Levene Statistic	*p*-Value
Absorbed Energy	0.965	0.78	1.12	0.36
Absorption Efficiency	0.961	0.73	0.98	0.41

**Table 2:** Average impact and residual velocities of epoxy composites reinforced with raffia fabric (ER10, ER20 and ER30) subjected to ballistic testing with 9 mm ammunition. Values are reported as mean ± standard deviation (n = 5).

Sample	V_i_ (m/s)	V_r_ (m/s)
ER10	433.67 ± 4.13	379.33 ± 2.48
ER20	433.07 ± 2.43	397.03 ± 2.12
ER30	434.08 ± 1.65	411.10 ± 1.57

**Table 3:** Equivalent ballistic limit (V_L,eq_) and absorbed energy (E_abs_) of epoxy composites reinforced with raffia fabric (ER10, ER20 and ER30) subjected to ballistic testing with 9 mm ammunition. Values are reported as mean ± standard deviation (n = 5).

Sample	V_L,eq_ (m/s)	E_abs_ (J)
ER10	210.14 ± 5.81	176.74 ± 9.71
ER20	172.95 ± 1.93	119.66 ± 2.67
ER30	139.17 ± 7.90	77.67 ± 8.96

**Table 4:** Absorbed energy (E_abs_), target mass, and specific energy absorption (SEA) of epoxy composites reinforced with raffia fabric at different fiber volume fractions.

Sample	Target Mass (g)	E_abs_ (J)	SEA (J/kg)
ER10	196.47	176.74	899.60
ER20	193.13	119.66	619.58
ER30	189.80	77.67	409.23

**Table 5:** Energy absorption efficiency, residual energy and moment reduction of epoxy composites reinforced with raffia fabric (ER10, ER20 and ER30) subjected to ballistic testing with 9 mm ammunition. Values are reported as mean ± standard deviation (n = 5).

Sample	Absorption Efficiency (%)	Residual Energy (J)	Momentum Reduction (Fraction)
ER10	23.48 ± 0.93	575.60 ± 7.55	0.1253 ± 0.0053
ER20	15.95 ± 0.26	630.60 ± 6.74	0.0832 ± 0.0014
ER30	10.30 ± 1.12	676.10 ± 5.17	0.0529 ± 0.0059

**Table 6:** Results of the analysis of variance (ANOVA) for the absorbed energy (E_abs_) of epoxy composites reinforced with raffia fabric in the proportions ER10, ER20 and ER30.

Source of Variation	SQ	df	MS	F	*p*
Between groups	24,740.6	2	12,370.3	204.7	<0.001
Within groups	725.4	12	60.45	-	-
Total	25,466.0	14	-	-	-

SQ—Sum of Squares; df—Degrees of Freedom; MS—Mean Square.

**Table 7:** Results of the Tukey multiple comparisons test (HSD) applied to the absorbed energy (E_abs_) of epoxy composites reinforced with raffia fabric (ER10, ER20 and ER30).

Comparison	Mean Difference (J)	Critical HSD (J)	Result
ER10 × ER20	57.08	13.10	Significant
ER10 × ER30	99.08	13.10	Significant
ER20 × ER30	41.99	13.10	Significant

**Table 8:** Analysis of variance (ANOVA) of the energy absorption efficiency of epoxy composites reinforced with raffia fabric (ER10, ER20 and ER30).

Source of Variation	SQ	df	MS	F	*p*
Between groups	436.2	2	218.1	299.5	<0.001
Within groups	8.72	12	0.73	-	-
Total	444.9	14	-	-	-

SQ—Sum of Squares; df—Degrees of Freedom; MS—Mean Square.

**Table 9:** Results of the Tukey multiple comparisons test (HSD) applied to the energy absorption efficiency of epoxy composites reinforced with raffia fabric (ER10, ER20 and ER30).

Comparison	Mean Difference (%)	Critical HSD (%)	Result
ER10 × ER20	7.53	1.43	Significant
ER10 × ER30	13.18	1.43	Significant
ER20 × ER30	5.65	1.43	Significant

**Table 10:** Comparative overview of ballistic performance parameters under 9 mm full-metal-jacket (FMJ) impact for the present raffia/epoxy composites and selected literature systems, considering incident velocity (V_i_), incident energy (E_i_), absorbed energy (E_abs_), and absorption efficiency (%E_abs_).

Material/System	Matrix	Reinforcement	Configuration	V_i_ (m/s)	E_i_ (J)	E_abs_ (J)	%E_abs_	Notes	Reference
Epoxy/Raffia (ER10)	Epoxy	Raffia fabric	Laminate (10 vol.%)	433.67	750.00	176.74	23.48	Best energy dissipation	This work
Epoxy/Raffia (ER20)	Epoxy	Raffia fabric	Laminate (20 vol.%)	433.07	750.00	119.66	15.95	Intermediate behavior	This work
Epoxy/Raffia (ER30)	Epoxy	Raffia fabric	Laminate (30 vol.%)	434.08	750.00	77.67	10.30	More rigid, less dissipative	This work
Veneer/Aramid hybrid (VAV2)	Epoxy (adhesive)	Densified veneer + aramid	V–A–V (2:1)	335.05	451.00	354.70	78.64	Hybrid high-performance armor	[23]
HDPE/Rattan (170 °C)	HDPE	Rattan fiber	Plate (30 vol.%)	324.30	420.68	57.55	13.68	Thermoplastic composite	[24]

## Data Availability

The original contributions presented in the study are included in the article; further inquiries can be directed to the corresponding author.
